# Suppression of MIR31HG affects the functional properties of thyroid cancer cells depending on the miR-761/MAPK1 axis

**DOI:** 10.1186/s12902-022-00962-3

**Published:** 2022-04-20

**Authors:** Shuwang Peng, Luyang Chen, Zhengtai Yuan, Shanshan Duan

**Affiliations:** 1 Department of Gastrointestinal and Thyroid and Vascular Surgery, The First Hospital of Hunan University of Chinese Medicine, Ward 22, 13th floor, Zhihe Building, No.95 Shaoshan Middle Road, Yuhua District, Changsha, 410000 Hunan, Province China; 2 Department of Ultrasound Imaging, The First Hospital of Hunan University of Chinese Medicine, Changsha, Hunan China

**Keywords:** Thyroid cancer, MIR31HG, miR-761, MAPK1, Functional properties

## Abstract

**Background:**

Thyroid cancer is the most prevalent endocrine malignancy. Long non-coding RNA (lncRNA) MIR31HG is abnormally expressed in thyroid cancer tissues. However, the precise, critical role of MIR31HG in thyroid cancer development remains unclear.

**Methods:**

MIR31HG, microRNA (miR)-761 and mitogen-activated protein kinase 1 (MAPK1) were quantified by quantitative real-time PCR (qRT-PCR) and immunoblotting. Cell viability, proliferation, apoptosis, invasion and migration abilities were evaluated by MTS, 5-Ethynyl-2′-Deoxyuridine (EdU), flow cytometry, transwell and wound-healing assays, respectively. Dual-luciferase reporter assays were used to validate the direct relationship between miR-761 and MIR31HG or MAPK1.

**Results:**

MIR31HG was overexpressed in human thyroid cancer, and its overexpression predicted poor prognosis. Suppression of MIR31HG impeded cell proliferation, invasion and migration, as well as promoted cell apoptosis in vitro, and diminished the growth of xenograft tumors in vivo. Mechanistically, MIR31HG targeted and regulated miR-761. Moreover, miR-761 was identified as a molecular mediator of MIR30HG function in regulating thyroid cancer cell behaviors. MAPK1 was established as a direct and functional target of miR-761 and MAPK1 knockdown phenocopied miR-761 overexpression in impacting thyroid cancer cell behaviors. Furthermore, MIR31HG modulated MAPK1 expression by competitively binding to miR-761 via the shared binding sequence.

**Conclusion:**

Our findings demonstrate that MIR31HG targets miR-761 to regulate the functional behaviors of thyroid cancer cells by upregulating MAPK1, highlighting a strong rationale for developing MIR31HG as a novel therapeutic target against thyroid cancer.

**Supplementary Information:**

The online version contains supplementary material available at 10.1186/s12902-022-00962-3.

## Highlights

(1) MIR31HG targeted and regulated miR-761.

(2) MAPK1 was a direct and functional target of miR-761.

(3) MIR31HG affected the functional behaviors of thyroid cancer cells by miR-761/MAPK1 axis.

**Supplementary Information:**

The online version contains supplementary material available at 10.1186/s12902-022-00962-3.

## Background

Because of the increased diagnostic imaging and the changes in risk factors, the incidence of thyroid cancer has been increasing, ranking in the 9th place of cancer incidence in 2018 [3]. Although thyroid cancer has a ~ 0.4% low mortality rate, it is the most prevalent endocrine malignancy, contributing to most deaths from endocrine malignant neoplasms [3, 18]. Vital players in the molecular pathology of thyroid cancer, including proteins and non-coding RNAs (ncRNAs), are under exploration at present [1, 5, 24]. Knowing the roles of these molecules can be beneficial for early diagnosis or even treatment of thyroid cancer.

As a heterogeneous kind of ncRNAs, long ncRNAs (lncRNAs) possess pivotal roles in gene expression during normal developmental and pathologic processes by means of diverse mechanisms, including post-transcriptional events [6, 7]. Particularly relevant in tumor, lncRNAs have established roles as anti-tumor factors and oncogenic drivers in every major cancer type via targeting microRNAs (miRNAs) and preventing their binding to mRNAs [2]. Studies in thyroid cancer models have provided evidence that dysregulated lncRNAs are crucial players in thyroid tumorigenesis [17] depending on the miRNA/mRNA networks [15, 23]. For instance, lncRNA MFI2-AS1 is overexpressed during thyroid tumorigenesis and contributes to thyroid carcinogenesis partially by pairing to miR-125a-5p to induce TRIAP1 [34]. Moreover, the promoting impact of lncRNA MALAT1 on thyroid tumorigenesis has been identified depending on the modulation of the miR-204/IGF2BP2 axis [32].

MIR31HG, a 2166 bp long lncRNA that is located at chromosome 9p21.3, has established different roles in human carcinogenesis [14, 29, 31]. Examples of MIR31HG as a potential tumor promoter include malignant melanoma and cervical carcinoma, where it is present at high levels [14, 29]. Conversely, elevated expression of MIR31HG hinders the growth and motility of hepatocellular carcinoma cells, highlighting its cancer suppressive activity [31]. Interestingly, overexpression of MIR31HG has recently been found in thyroid cancer tissue samples [21]. Nevertheless, no studies documented whether different expression of MIR31HG is causally implicated in thyroid carcinogenesis. Therefore, we sought to elucidate the precise action of MIR31HG in the functional behaviors of thyroid cancer cells and its regulation through MIR31HG-mediated the activity of competing endogenous RNA (ceRNA).

## Materials and methods

### Human tissue specimens and cell lines

The study cohort comprised a consecutive series of 29 patients with thyroid cancer resected for cure at the First Hospital of Hunan University of Chinese Medicine from April 2015 to January 2016. With the formal consent form, we collected primary thyroid cancer specimens and adjacent noncancerous thyroid tissues from these patients by surgery before treatment and preserved them following the requirements of the experiments. Patients with other cancers, cardiovascular diseases, severe metabolic diseases or severe infections were excluded from this study. None of the patients received any conventional treatment before surgery, and patients with complete medical information were included in the study. Frozen specimens were used for MIR31HG, miR-761 and mitogen-activated protein kinase 1 (MAPK1) quantification as below, and formalin-fixed paraffin-embedded specimens were used for Ki67 staining by immunohistochemistry with a rabbit anti-Ki67 antibody (PA5–19462, Invitrogen, Basel, Switzerland) at a 1:200 dilution as described elsewhere [12]. We collected the follow-up data of patients from the Registry of our hospital. Experimental protocol for human specimen collection and use was approved by the Ethics Committee of the First Hospital of Hunan University of Chinese Medicine. The study was conducted in accordance with the Declaration of Helsinki (as revised in 2013).

We obtained human SW579 (thyroid squamous cell carcinoma) and TPC-1 (papillary thyroid cancer) cells from Procell (Wuhan, China) and HTH83 (anaplastic thyroid cancer) and Nthy-ori 3–1 (normal thyroid epithelial) cells from Bnbio (Beijing, China). We propagated the cells in the following media from Gibco (Paisley, UK): RPMI-1640 for TPC-1 and HTH83 cells, Leibovitz’s L-15 for SW579 cells and DMEM for Nthy-ori 3–1 cells. All cells were grown at 5% CO_2_ at 37 °C in media plus 10% FBS and 1% streptomycin/penicillin (all from Euroclone, Milano, Italy).

### RNA preparation and quantitative real-time PCR (qRT-PCR)

To extract total RNA from cultured cells and tissue specimens, we applied a RiboPure™ RNA Kit (Invitrogen) based on the manufacturing recommendations. To prepare nuclear and cytoplasmic RNA of SW579 and TPC-1 cells, we employed a Cytoplasmic & Nuclear RNA Purification Kit from Norgen Biotek (Thorold, ON, Canada). For MIR31HG, MAPK1 mRNA and GAPDH mRNA analyses, cDNA was produced in a 10 μL of reaction volume containing 1 μg of RNA, 0.3 μg of oligo (dT)_18_ primer (TaKaRa, Dalian, China) and 100 U of M-MLV reverse transcriptase (Promega, Sydney, Australia); the cDNA in a 25 μL of reaction mixture was then amplified using VeriQuest SYBR Green (Affymeterix, Schwerte, Germany) and 10 pmol of each forward and reverse primers (Supplement Table 1). For miR-761 quantification, miScript RT Kit (Qiagen, Courtaboeuf, France), miScript SYBR Green PCR Kit (Qiagen) and specific primer for miR-761 (Supplement Table 1) were used in this study. A housekeeping gene β-actin or U6 was used to correct for differences in the amount of RNA in each sample. For relative quantification, we adopted the 2^-ΔΔCt^ expression formula.

### Plasmid, siRNA, miRNA mimic or inhibitor transfection

To silence MIR31HG in cells, we purchased Silencer® Select siRNA for MIR31HG (si-MIR31HG) from Thermo Fisher Scientific (Milan, Italy). To express miR-761 in cells, we transfected mirVana® miRNA mimic for miR-761 (Thermo Fisher Scientific) into cells. To knock down available miR-761 in cells, we obtained mirVana® miRNA Inhibitor for miR-761 (anti-miR-761) from Thermo Fisher Scientific and transfected it into cells. The siRNA-scramble (si-NC), miRNA-scramble (miR-NC) and inhibitor-scramble (anti-miR-NC) served as non-specific controls. To express MIR31HG or MAPK1 in cells, we cloned MIR31HG full-length sequence or MAPK1 coding sequence (lacking 3’UTR), synthesized by Abiocenter (Beijing, China), into the pcDNA3.1 vector (Thermo Fisher Scientific).

For transient transfection, we seeded SW579 and TPC-1 cells (100,000 cells/well) in 12-well culture dishes before transfection using Lipofectamine 2000 (Thermo Fisher Scientific) with plasmid (300 ng), miRNA mimic/inhibitor (50 nM) or siRNA (150 nM). We harvested the cells after 48 h for expression analysis and functional experiment.

### MTS assay for cell viability

We plated SW579 and TPC-1 cells after the appropriate transfection into 96-well culture dishes at 5000 cells per well and maintained them overnight at 37 °C. Subsequently, each well received 10 μL of MTS (3-(4,5-dimethylthiazol-2-yl)-5-(3-carboxymethoxyphenyl)-2-(4-sulfophenyl)-2H-tetrazolium) solution as recommended by the manufacturers (BestBio, Shanghai, China). Following a 4-h incubation at 37 °C, we measured the absorption by spectrophotometry (TECAN, Männedorf, Switzerland) at 490 nm.

### 5-Ethynyl-2′-Deoxyuridine (EdU) assay for cell proliferation

We incubated SW579 and TPC-1 cells after the appropriate transfection with EdU solution (50 μM, Yeasen, Shanghai, China) for 2 h before EdU staining with Apollo 488 (Ribobio, Guangzhou, China). Following the nuclei staining with 4′,6-diamidino-2-phenylindole (DAPI, Solarbio, Beijing, China), we analyzed the proliferation rate as the percentage of EdU positive cells (green) relative to total cells (blue) using a fluorescence microscope (Olympus, Hamburg, Germany).

### Flow cytometry for cell apoptosis

We stained SW579 and TPC-1 cells (500,000 cells per sample) after the appropriate transfection with 25 μg/mL of Annexin V-FITC (Yeasen) and 50 μg/mL of propidium iodide (PI, MedChemExpress, Tokyo, Japan) in PBS. About 10,000 events were acquired in a FACS Aria III flow cytometer (BD Biosciences, North Ryde, Australia) and analyzed for percent cells undergoing apoptosis (Annexin V^+^/PI^−^ and Annexin V^+^/PI^+^) with AccuriC6 software from BD Biosciences.

### Transwell invasion assay

For evaluation of cell migration, we used 24-well, 6.5 mm internal diameter transwell plates (BD Biosciences) with Matrigel-coated membranes (8 μm pore size) separating the 2 chambers. SW579 and TPC-1 cells after the appropriate transfection were seeded in non-serum media in the upper chamber at 50,000 cells per well. 10% FBS medium served as chemoattractant in the lower chamber. 24 h post-seeding, non-invading cells were removed and invaded cells were fixed with methanol. After crystal violet (0.1%) staining, images were captured by Olympus CKX41 at 100× magnification and registered using the getIT software (Olympus).

### Wound-healing assay for cell migration

We plated SW579 and TPC-1 cells after the appropriate transfection into 6-well dishes (500,000 cells/well) and cultured them until ~ 80% confluence. We created a scratch wound using a sterile plastic 200 μL pipette tip. Cell migration was photographed and measured by microscopy at 10× magnification.

### Immunoblotting

For immunoblotting under standard methods [13], we isolated total protein using cold RIPA lysis buffer (Solarbio) from cultured cells and tissue specimens homogenized by a tissue grinder and separated it by SDS-PAGE, followed by blotting onto nitrocellulose membranes (Millipore, Oxen, UK). Antibodies against MAPK1 (sc-16,472, Santa Cruz Biotechnology, Heidelberg, Germany), Cleaved-caspase-3 (ab32042, Abcam, Cambridge, UK), matrix metalloproteinase 9 (MMP9, ab137867, Abcam) and GAPDH (ab8245, Abcam) were used, which were visualized with IgG secondary antibody conjugated to HRP (ab97051 and ab97023, Abcam) and enhanced chemiluminescence (Millipore). The images of the original blots were shown in Supplementary information file (the blots were cut before hybridisation with antibodies).

### Bioinformatics

For prediction of miRNAs that potentially bind to MIR31HG, we used the computer algorithm LncBase Predicted v.2 (http://carolina.imis.athena-innovation.gr/diana_tools/web/index.php?r=lncbasev2/index-predicted). For prediction of miRNA-binding sites in human 3’UTRs, we employed the target prediction tool ENCORI (http://starbase.sysu.edu.cn/).

### Dual-luciferase reporter assay

We obtained the fragments of MIR31HG and MAPK1 3’UTR encompassing the predicted miR-761 complementary sequence or mutated complementary seed region from Abiocenter and inserted them into a pmirGLO vector (Promega, Vienna, Austria). For luciferase assay, transfection experiments were done using Lipofectamine 2000 in SW579 and TPC-1 cells (100,000 cells/well) in 12-well dishes. The transfection mixture consisted of 200 ng of individual reporter construct and 30 nM of miRNA mimic. We harvested the cells at 48 h post-transfection for luciferase assay by Dual-luciferase Assay System (Promega).

### Generation of stable MIR31HG depletion cell line

To generate TPC-1 cells stably expressing shRNA-MIR31HG (sh-MIR31HG), we obtained the lentivirus coding sh-MIR31HG from VectorBulider (Guangzhou, China). A shRNA-scramble (sh-NC, VectorBulider) served as a non-specific control. We transduced TPC-1 cells with the lentivirus and puromycin-selected to obtain stable cell lines.

### Mouse xenografts

All mouse studies adhered to protocols approved by the Animal Care and Use Committee of the First Hospital of Hunan University of Chinese Medicine. For formation of subcutaneous xenografts, we subcutaneously injected TPC-1 cells stably expressing sh-MIR31HG or sh-NC (5000,000 cells/injection) in 150 μL PBS into the right flanks of male BALB/c nude mice aged 8-week (Beijing Vital River Laboratory Animal Technology Co., Ltd., Beijing, China). Measurement of xenograft growth was periodically conducted, and tumor volume was estimated using the formula 0.5 × (length × width^2^). All mice were sacrificed by anesthetic overdose on day 22, and the xenografts were harvested for weight and expression analysis. Sections of paraffin-embedded xenografts were processed by immunohistochemistry by staining with anti-Ki67 antibody (PA5–19462) as described elsewhere [12].

### Statistical analysis

Unless otherwise noted, all experiments were repeated at least three times (in triplicate), with results presented as mean ± standard deviation. For analysis of data normal distribution, we used Shapiro-Wilk normality test. Two group means were analyzed using a Student’s *t*-test (two-tailed) and multiple group means were compared by two-way ANOVA, followed by the Tukey’s post hoc test. For analysis of overall survival of these patients, we used Kaplan-Meier method and log-rank test (for significance). For analysis of correlations of variables in tumor specimens, we employed Pearson’s rank correlation coefficient. Significance was defined as *P* < 0.05.

## Results

### Overexpression of MIR31HG in thyroid cancer

**Fig. 1:**
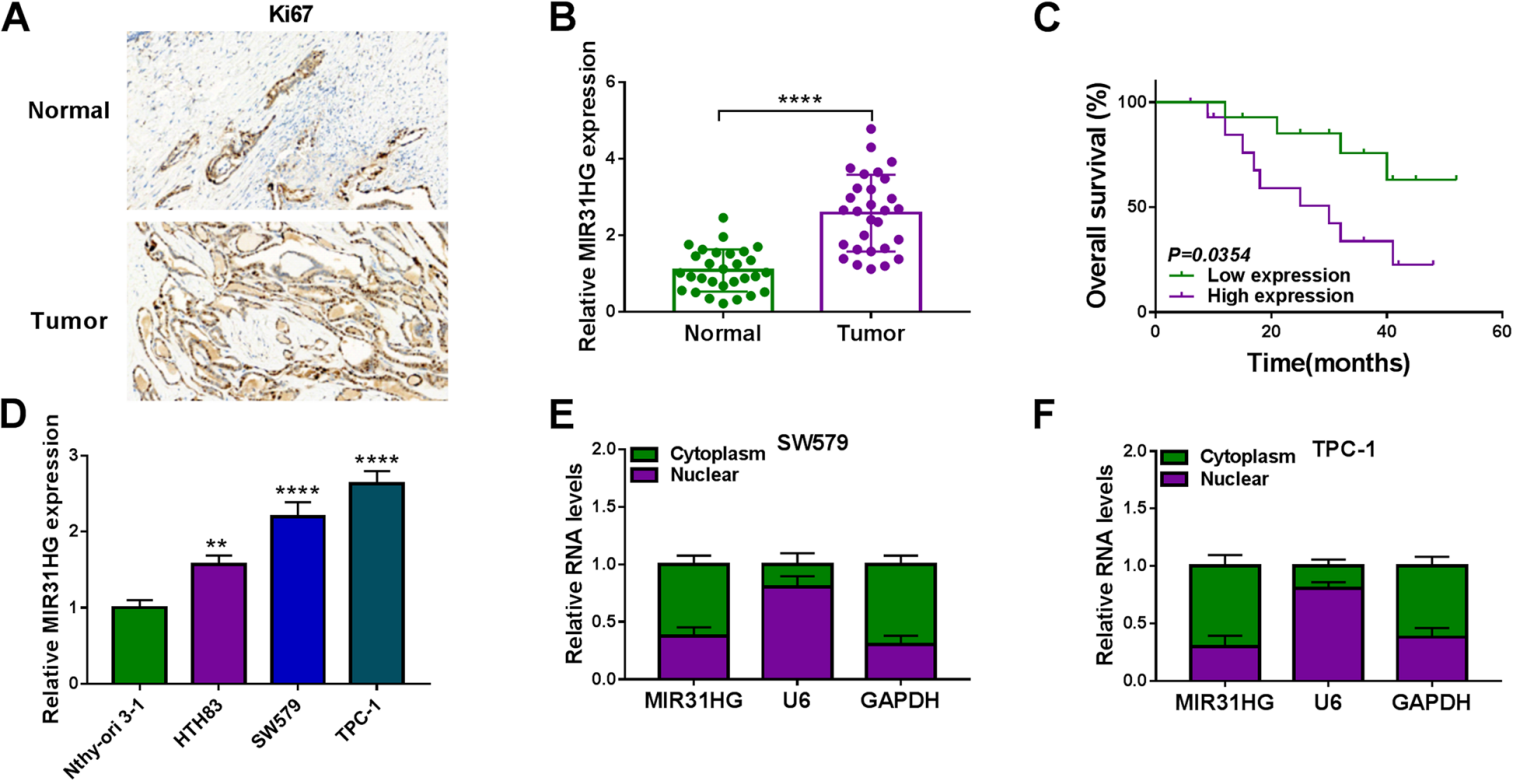
MIR31HG is highly expressed in thyroid cancer. **A** Immunohistochemical staining of Ki67 in primary tumor samples and matched normal thyroid samples from the same patients (*n* = 5). **B** Relative MIR31HG expression as measured by qRT-PCR in primary tumor samples and matched normal thyroid samples from the same patients (*n* = 29), normalized to β-actin expression. **C** Kaplan-Meier survival curves of these patients with high MIR31HG expression (*n* = 13) and low MIR31HG expression (*n* = 16). The *p* value was from a long-rank test. **D** Relative MIR31HG expression in HTH83, SW579, TPC-1, and Nthy-ori 3–1 cells by qRT-PCR, normalized to β-actin expression. **E** and **F** The subcellular localization of MIR31HG in SW579 and TPC-1 cells was determined by subcellular fractionation analysis. U6 and GAPDH served as controls. **P < 0.01, ****P < 0.0001

To elucidate the clinical significance of MIR31HG in thyroid cancer, we recruited a cohort of 29 thyroid cancer cases and collected primary cancer samples and matched thyroid samples. To confirm successful tumor collection, we performed immunohistochemical analysis for Ki67 staining in these samples. Expectedly, collected tumor samples had significantly more cells stained for Ki67 staining than the matched normal controls (Fig. 1A). Analysis of MIG31HG expression in primary cancer samples to paired thyroid samples, using qRT-PCR of total RNA preparations, showed that MIR31HG expression was at high levels in tumor tissues (Fig. 1B). To test the association between MIR31HG level and prognosis of these patients, we employed the Kaplan-Meier survival analysis and long-rank test. When these patients were divided into two groups (high MIR31HG expression group and low MIR31HG expression group) according the mean of MIR31HG level, we found that patients with low MIR31HG expression had a better survival rate than those with high MIR31HG level (Fig. 1C). Moreover, overexpression of MIR31HG in thyroid cancer cells (HTH83, SW579, and TPC-1) was validated by qRT-PCR compared to normal Nthy-ori 3–1 cells (Fig. 1D). Additionally, MIR31HG predominantly localized to the cytoplasm of SW579 and TPC-1 cells, which was ascertained by subcellular localization assay (Fig. 1E and F).

### Effects of MIR31HG suppression on cell proliferation, apoptosis, invasion and migration in vitro

**Fig. 2:**
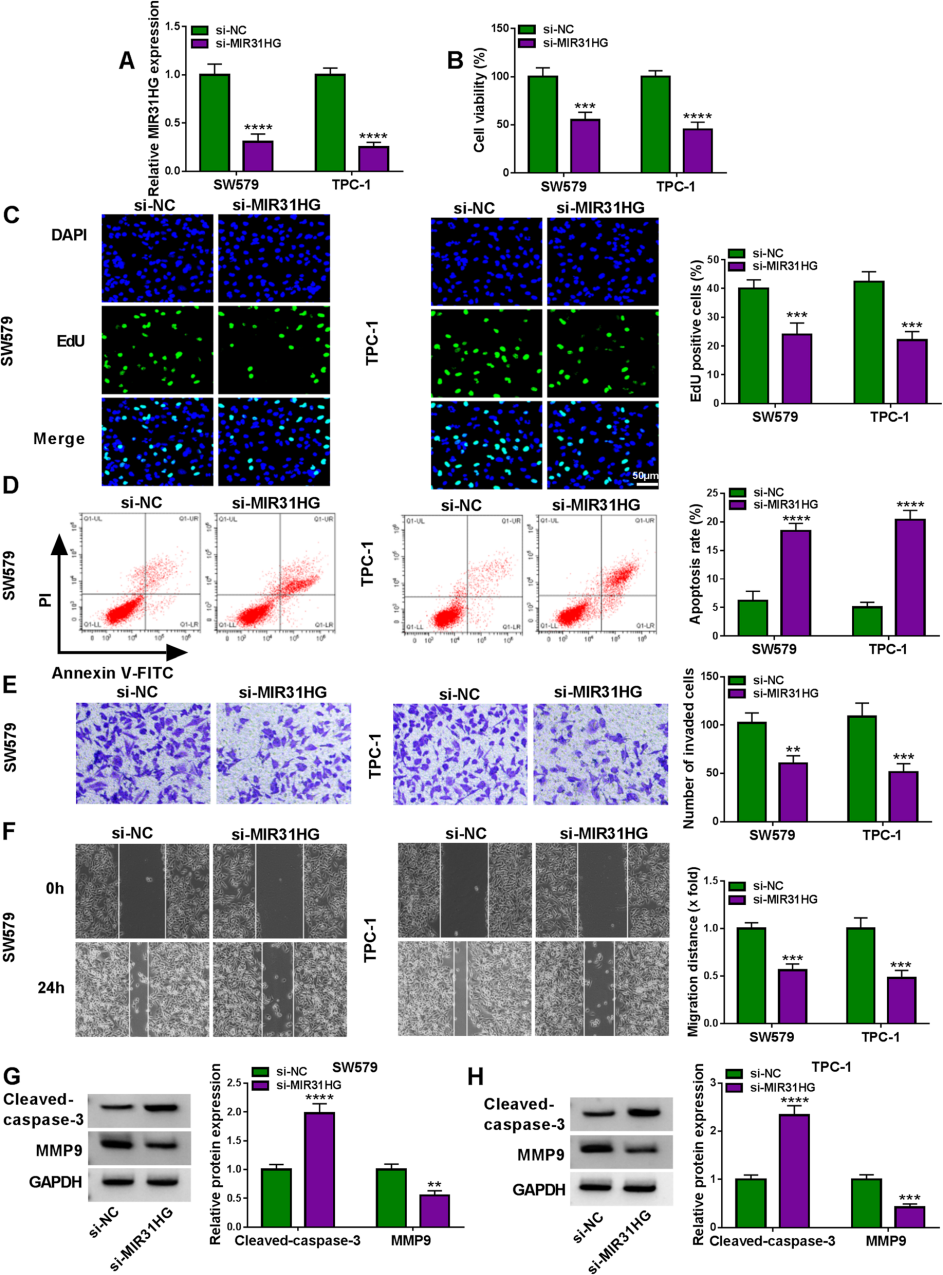
MIR31HG depletion affects cell proliferation, apoptosis, invasion and migration in vitro. **A** Effect of si-MIR31HG or si-NC introduction on MIR31HG expression in SW579 and TPC-1 cells gauged by qRT-PCR, normalized to β-actin expression. **B** Effect of si-MIR31HG or si-NC transfection on cell viability determined by MTS assay. **C** Effect of si-MIR31HG or si-NC transfection on the proliferation rate of SW579 and TPC-1 cells, based on the staining with EdU and DAPI. Scale bars, 50 μm. **D** Representative images depicting a cell apoptosis assay and apoptosis rate of si-MIR31HG-transfected or si-NC-introduced SW579 and TPC-1 cells by flow cytometry, based on the Annexin V/PI staining. **E** Effect of si-MIR31HG or si-NC transfection on the invasion rate of SW579 and TPC-1 cells as measured by transwell assay. **F** Representative pictures showing the migration ability of si-MIR31HG-transfected or si-NC-introduced SW579 and TPC-1 cells as evaluated by wound-healing assay. **G** and **H** The protein levels of Cleaved-caspase-3 and MMP9 in si-MIR31HG-transfected or si-NC-introduced SW579 and TPC-1 cells by immunoblotting. GAPDH served as a loading control for relative protein quantification. **P < 0.01, ***P < 0.001, ****P < 0.0001

We then undertook to characterize the functional consequences of MIR31HG inhibition on cell functional properties in vitro. For this purpose, we generated depletion of MIR31HG in SW579 and TPC-1 cells using siRNA-MIR31HG (si-MIR31HG). When si-MIR31HG transfection into the two cell lines, MIR31HG expression was remarkably decreased (Fig. 2A), demonstrating the effectiveness of the inhibition. MIR31HG depletion resulted in suppressed cell viability (Fig. 2B) and proliferation rate (Fig. 2C) in both cell lines. Conversely, MIR31HG depletion strongly accelerated the apoptosis rate of SW579 and TPC-1 cells (Fig. 2D). Furthermore, SW579 and TPC-1 cells with MIR31HG inhibition exhibited reduced cell invasion (Fig. 2E) and migration (Fig. 2F) rates compared with the si-NC group. In addition, MIR31HG inhibition in SW579 and TPC-1 cells led to a clear elevation of apoptosis-related protein Cleaved-caspase-3 expression and a strong downregulation in the level of motility-related protein MMP9 (Fig. 2G and H). All these results indicate that MIR31HG inhibition impedes cell proliferation, invasion and migration and promotes cell apoptosis.

### MIR31HG regulates miR-761 expression

**Fig. 3:**
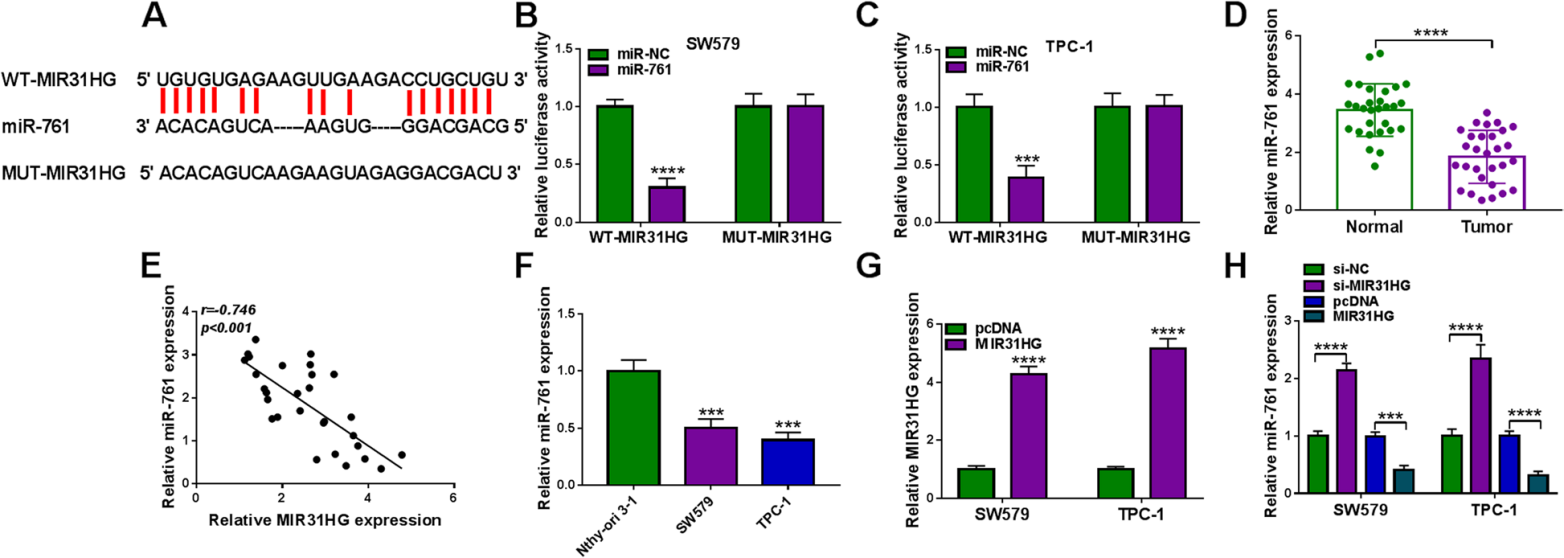
MIR31HG regulates miR-761 expression by directly pairing to miR-761. **A** MIR31HG segment harboring the putative miR-761 pairing region, mature miR-761 sequence, and the mutations in the predicted miR-761 pairing site. **B** and **C** Effects of miR-761 on the activity of the luciferase reporter construct containing the wild-type (WT) or mutated (MUT) miR-761 pairing site in SW579 and TPC-1 cells. **D** Relative miR-761 expression as gauged by qRT-PCR in primary tumor samples and matched normal thyroid samples from the same patients (*n* = 29), normalized to U6 expression. **E** Expression correlation between miR-761 and MIR31HG in primary tumor samples (*n* = 29). Correlation value (r) was Pearson’s rank correlation coefficient. **F** Relative miR-761 expression in SW579, TPC-1, and Nthy-ori 3–1 cells measured by qRT-PCR, normalized to U6 expression. **G** Effects of MIR31HG expressing plasmid introduction and pcDNA control transfection on MIR31HG expression in SW579 and TPC-1 cells by qRT-PCR, normalized to β-actin expression. **H** Effects of the transfection of si-MIR31HG, si-NC, MIR31HG expressing plasmid, and pcDNA control plasmid on miR-761 expression measured by qRT-PCR, normalized to U6 expression. ***P < 0.001, ****P < 0.0001

While investigating the molecular mechanism by which MIR31HG affects cell functional properties, we observed that MIR31HG harbors a putative miR-761 binding site (LncBase Predicted v.2, Fig. 3A). The direct relationship between MIR31HG and miR-761 was verified by dual-luciferase reporter assays. Co-transfection of MIR31HG luciferase reporter and miR-761 mimic into SW579 and TPC-1 cells caused lower luciferase activity than the cells co-transfected with miR-NC mock (Fig. 3B and C). Site-directed mutant of the miR-761 target sequence strongly abrogated the effect of miR-761 on luciferase activity (Fig. 3B and C). Intriguingly, in thyroid cancer samples, we found a striking reduction of miR-761 expression (Fig. 3D) and a significant inverse correlation between miR-761 and MIR31HG expression (Fig. 3E). Moreover, qRT-PCR assay indicated that miR-761 was downregulated in SW579 and TPC-1 cells compared to normal Nthy-ori 3–1 cells (Fig. 3F). The ability of MIR31HG to influence miR-761 expression was examined by qRT-PCR. To address this, we manipulated MIR31HG expression using si-MIR31HG and a MIR31HG expressing plasmid. When the expressing construct was transfected into the two cancer cells, MIR31HG level was augmented by ~ 4-fold for SW579 cells and ~ 5-fold for TPC-1 cells (Fig. 3G), validating the MIR31HG upregulation efficacy of the construct. As would be expected, in SW579 and TPC-1 cells, MIR31HG inhibition markedly elevated the expression of miR-761, and MIR31HG overexpression exerted an opposite effect (Fig. 3H). Collectively, these data indicate that MIR31HG regulates miR-761 expression through a specific binding site.

### The effects of MIR31HG suppression is partly dependent on increased abundance of miR-761

**Fig. 4:**
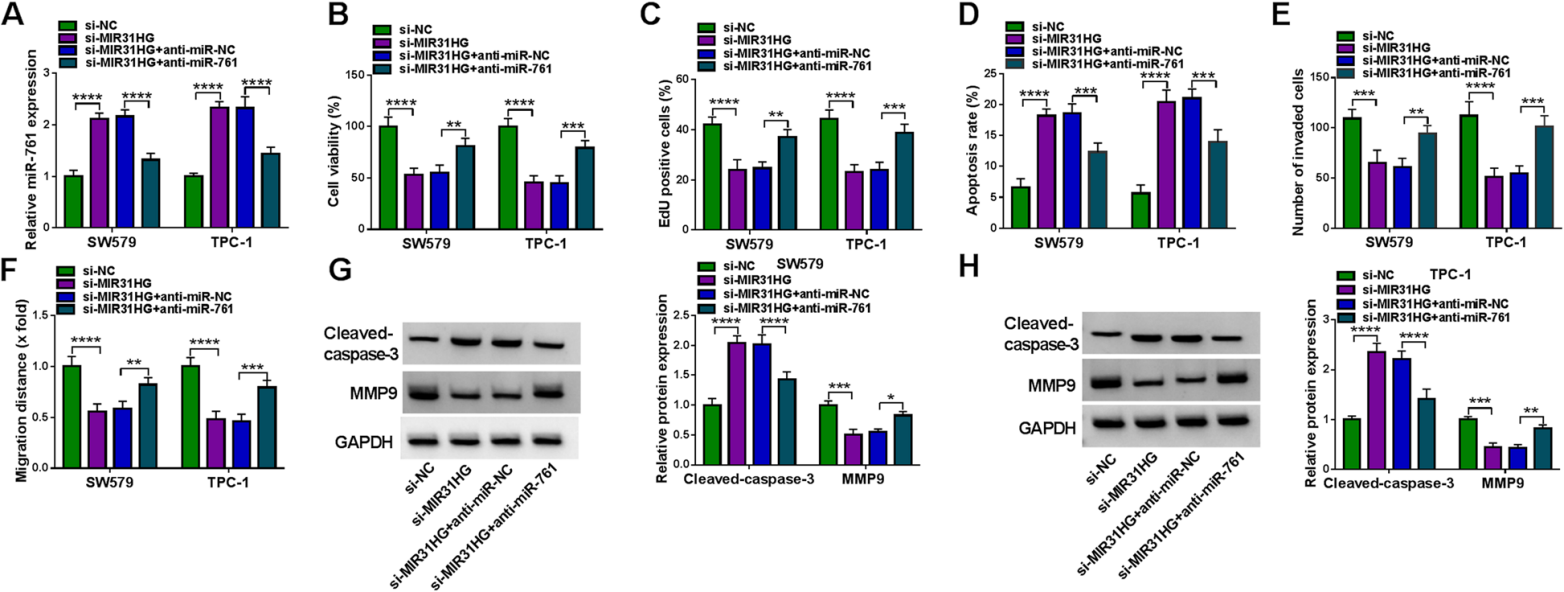
MIR31HG function is partly dependent on miR-761. **A** Effects of the transfection of anti-miR-761 + si-MIR31HG, anti-miR-NC + si-MIR31HG, si-MIR31HG or si-NC on miR-761 expression in SW579 and TPC-1 cells, normalized to U6 expression. **B** Effects of the indicated transfections on the viability of SW579 and TPC-1 cells by MTS assay. **C** Effects of the indicated transfections on the proliferation of SW579 and TPC-1 cells, based on the staining with EdU and DAPI. **D** Effects of the indicated transfections on the apoptosis of SW579 and TPC-1 cells, based on the Annexin V/PI staining. **E** Transwell assay for the invasion ability of SW579 and TPC-1 cells after the indicated transfections. **F** Wound-healing assay for the migration rate of SW579 and TPC-1 cells after the indicated transfections. **G** and **H** Representative immunoblotting analysis showing the protein levels of Cleaved-caspase-3 and MMP9 in SW579 and TPC-1 cells after the indicated transfections, with GAPDH as a loading control for relative protein quantification. *P < 0.05, **P < 0.01, ***P < 0.001, ****P < 0.0001

To determine whether miR-761 elevation is responsible for the effects of MIR31HG inhibition on cell functional properties, we set out to analyze the consequences of miR-761 reduction in SW579 and TPC-1 cells with MIR31HG inhibition. Transfection of miR-761 inhibitor (anti-miR-761), but not the scrambled control, strikingly lessened miR-761 expression in MIR31HG-silenced SW579 and TPC-1 cells (Fig. 4A). Notably, miR-761 reduction counteracted MIR31HG inhibition-driven cell viability (Fig. 4B) and proliferation (Fig. 4C) defects compared to the control group. Moreover, miR-761 reduction suppressed cell apoptosis induced by depletion of MIR31HG (Fig. 4D). Downregulation of miR-761 also abolished MIR31HG knockdown-mediated repression of invasion (Fig. 4E) and migration (Fig. 4F) of SW579 and TPC-1 cells. Our immunoblotting analysis also revealed that reduced miR-761 expression significantly abated the influence of MIR31HG inhibition on Cleaved-caspase-3 and MMP9 expression levels in both cell lines (Fig. 4G and H). These findings together demonstrate that the effects of MIR31HG inhibition on SW579 and TPC-1 cells depends, at least in part, on miR-761 abundance increase.

### MAPK1 is a direct and functional target of miR-761

**Fig. 5:**
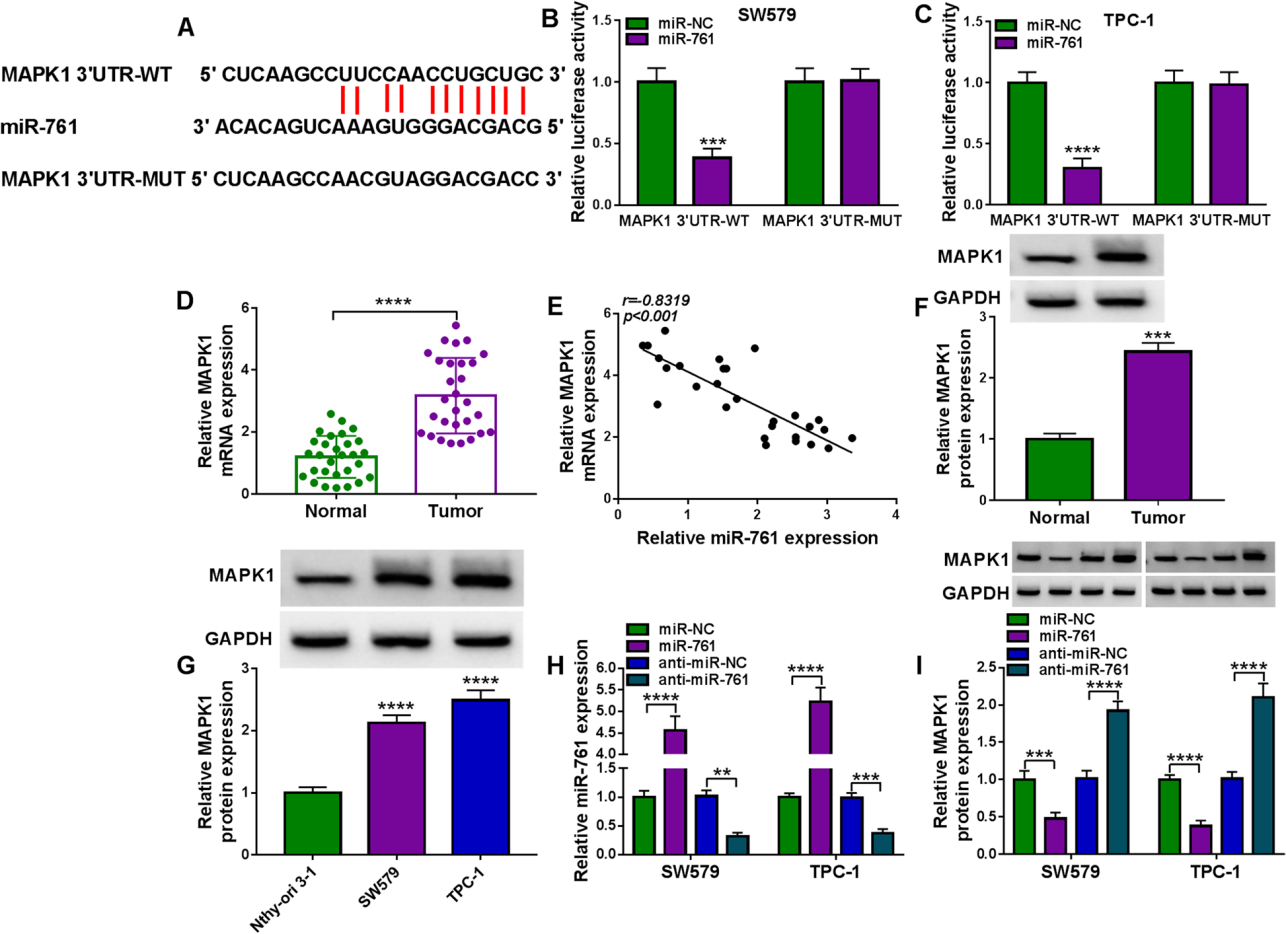
MiR-761 targets MAPK1. **A** MAPK1 3’UTR fragment encompassing the putative miR-761 pairing sequence, mature miR-761 sequence, and the mutations in the predicted miR-761 seed region. **B** and **C** Effects of miR-761 on the activity of the luciferase reporter construct containing the wild-type (WT) or mutated (MUT) miR-761 pairing site in SW579 and TPC-1 cells. **D** Relative MAPK1 mRNA expression gauged by qRT-PCR in primary tumor samples and matched normal thyroid samples from the same patients (*n* = 29), normalized to β-actin expression. **E** Expression correlation between MAPK1 mRNA and miR-761 in primary tumor samples (*n* = 29). Correlation value (r) was Pearson’s rank correlation coefficient. **F** Expression of MAPK1 protein in primary tumor samples and matched normal thyroid samples from the same patients (*n* = 5) by immunoblotting, with GAPDH as a loading control for expression quantification. **G** Relative MAPK1 protein expression in SW579, TPC-1, and Nthy-ori 3–1 cells measured by immunoblotting, with GAPDH as a loading control. **H** Effects of anti-miR-761, anti-miR-NC, miR-761 mimic and miR-NC control on miR-761 expression measured by qRT-PCR, normalized to U6 expression. **I** Effects of anti-miR-761, anti-miR-NC, miR-761 mimic and miR-NC control on relative MAPK1 protein expression gauged by immunoblotting, with GAPDH as a loading control for expression quantification

To identify the downstream targets of miR-761, we employed the target prediction tool ENCORI. The algorithm predicted a putative pairing site for miR-761 in the 3’UTR of MAPK1 (Fig. 5A), suggesting that miR-761 may target MAPK1. To experimentally verify this possibility, we adopted luciferase assays using two reporter constructs (MAPK1 3’UTR reporter and the mutant 3’UTR reporter). Transfection of miR-761 mimic decreased luciferase activity in lysates of SW579 and TPC-1 cells transfected with MAPK1 3’UTR reporter but had no effect on the 3’UTR construct carrying the mutated miR-761 complementary sequence (Fig. 5B and C). Furthermore, a remarkable upregulation of MAPK1 mRNA in thyroid cancer samples was validated by qRT-PCR (Fig. 5D). The striking inverse correlation between the levels of MAPK1 mRNA and miR-761 in thyroid cancer samples further supported the targeting of MAPK1 by miR-761 (Fig. 5E). Our immunoblotting analysis also showed a significant overexpression of MAPK1 protein in thyroid cancer samples and cells compared with the matched controls (Fig. 5F and G).

**Fig. 6:**
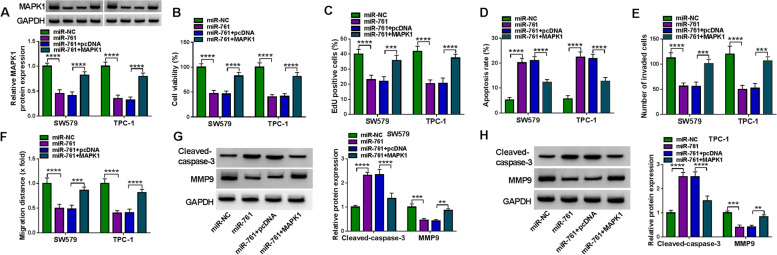
MAPK1 is a functionally downstream effector of miR-761. **A** Effects of the transfection of miR-761 mimic+MAPK1 expressing construct, miR-761 mimic+pcDNA control, miR-761 mimic and miR-NC mock on MAPK1 protein expression in SW579 and TPC-1 cells, with GAPDH as a loading control for expression quantification. **B** MTS assay of the viability of SW579 and TPC-1 cells after the indicated transfections. **C** Proliferation rate of SW579 and TPC-1 cells after the indicated transfections, based on the staining with EdU and DAPI. **D** Apoptosis rate of SW579 and TPC-1 cells after the indicated transfections, based on the Annexin V/PI staining. **E** Effects of the indicated transfections on the invasion ability of SW579 and TPC-1 cells determined by transwell assay. **F** Effects of the indicated transfections on the migration rate of SW579 and TPC-1 cells examined by wound-healing assay. **G** and **H** Immunoblotting of the protein levels of Cleaved-caspase-3 and MMP9 in SW579 and TPC-1 cells after the indicated transfections, with GAPDH as a loading control for relative protein quantification. **P < 0.01, ***P < 0.001, ****P < 0.0001

To further validate the regulation of miR-761 on MAPK1 expression, we manipulated miR-761 expression by transfecting SW579 and TPC-1 cells with miR-761 mimic and anti-miR-761. Transfection of miR-761 mimic increased miR-761 expression, while anti-miR-761 introduction exhibited an opposite effect (Fig. 5H). Expectedly, we observed that anti-miR-761-transfected SW579 and TPC-1 cells showed increased expression of endogenous MAPK1 protein (Fig. 5I). Moreover, mimic-mediated upregulation of miR-761 resulted in decreased levels of MAPK1 protein (Figs. 5I and 6A) and hindered cell viability (Fig. 6B) and proliferation (Fig. 6C), as well as reduced apoptosis (Fig. 6D), and suppressed invasion (Fig. 6E) and migration (Fig. 6F). Finally, SW579 and TPC-1 cells expressing miR-761 exhibited higher levels of Cleaved-caspase-3 protein and lower expression of MMP9 protein compared with the control group (Fig. 6G and H).

We further elucidated whether downregulation of MAPK1 protein expression may provide an explanation for the alteration of cell functional properties following miR-761 upregulation. For this purpose, we overexpressed MAPK1 in SW579 and TPC-1 cells after transfection by miR-761 mimic using a MAPK1 expressing construct that lacks the 3’UTR of MAPK1-encoding mRNA. Co-transfection of this construct clearly abated miR-761 elevation-driven reduction of MAPK1 protein level of SW579 and TPC-1 cells (Fig. 6A). Indeed, restoration of MAPK1 expression in SW579 and TPC-1 cells strongly abrogated miR-761-mediated cell viability reduction (Fig. 6B), proliferation repression (Fig. 6C), apoptosis increase (Fig. 6D), invasion reduction (Fig. 6E), migration inhibition (Fig. 6F) and the alteration of Cleaved-caspase-3 and MMP9 expression levels (Fig. 6G and H) compared to the controls. In summary, MAPK1 seems to be a crucial downstream effector of miR-761.

### MIR31HG is a post-transcriptional regulator of MAPK1 expression through miR-761

**Fig. 7:**
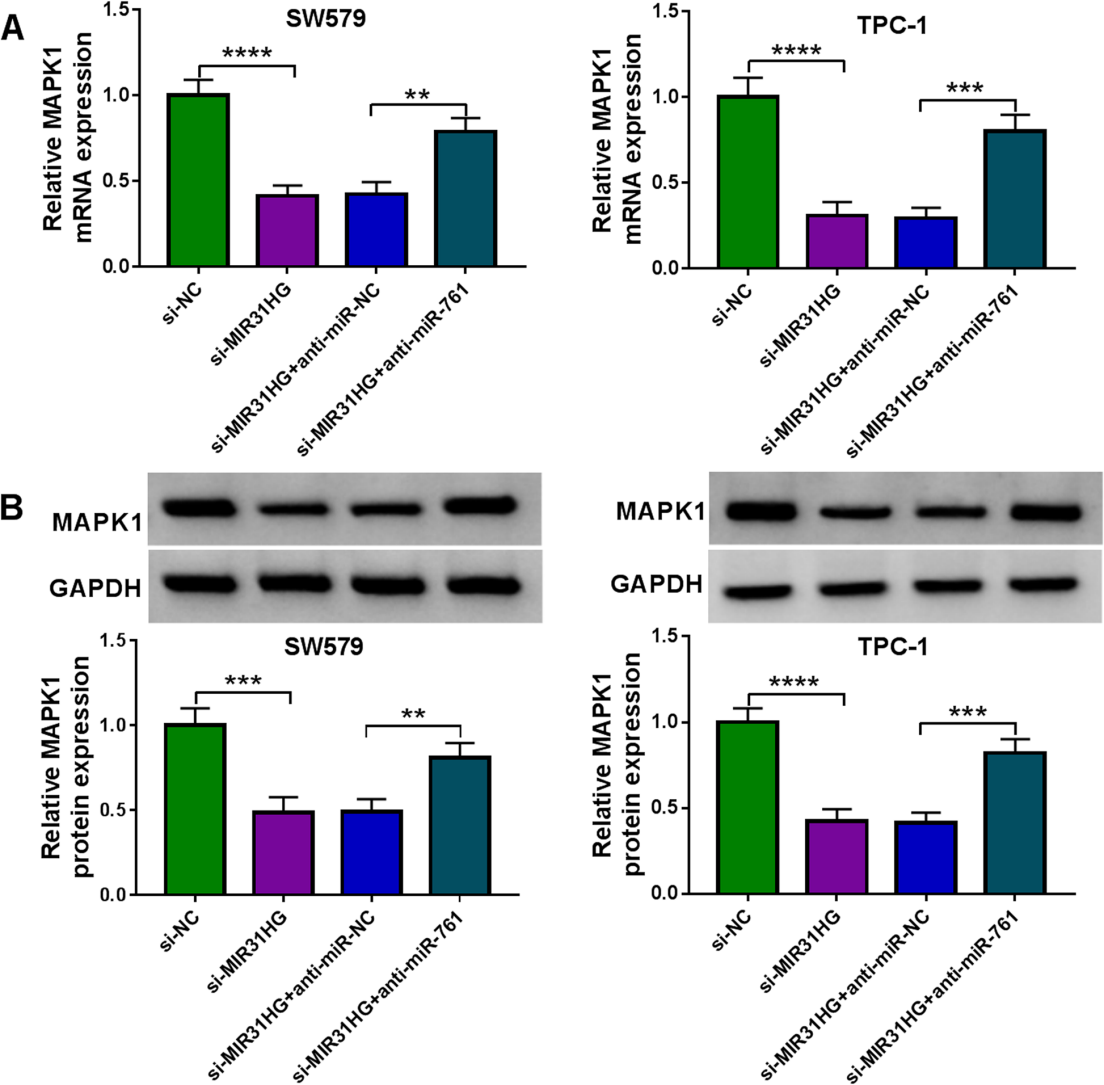
MIR31HG targets miR-761 to regulate MAPK1 expression. **A** Effects of the transfection of anti-miR-761 + si-MIR31HG, anti-miR-NC + si-MIR31HG, si-MIR31HG and si-NC on MAPK1 mRNA expression as measured by qRT-PCR, normalized to β-actin expression. **B** Effects of the transfection of anti-miR-761 + si-MIR31HG, anti-miR-NC + si-MIR31HG, si-MIR31HG and si-NC on MAPK1 protein expression as detected by immunoblotting, with GAPDH as a loading control for relative protein quantification. **P < 0.01, ***P < 0.001, ****P < 0.0001

Having demonstrated that MIR31HG targets miR-761 that targets MAPK1, we next tested whether MIR31HG can affect MAPK1 expression. Notably, we found that MIR31HG-silenced SW579 and TPC-1 cells exhibited lower levels of MAPK1 mRNA and protein compared with the same cells expressing a scrambled control sequence (Fig. 7A and B). However, this effect was significantly abolished by anti-miR-761 transfection (Fig. 7A and B), indicating that MIR31HG involves the modulation of MAPK1 expression through competitively binding to miR-761 by the shared binding sequence.

### MIR31HG suppression diminishes the growth of xenograft tumors

**Fig. 8:**
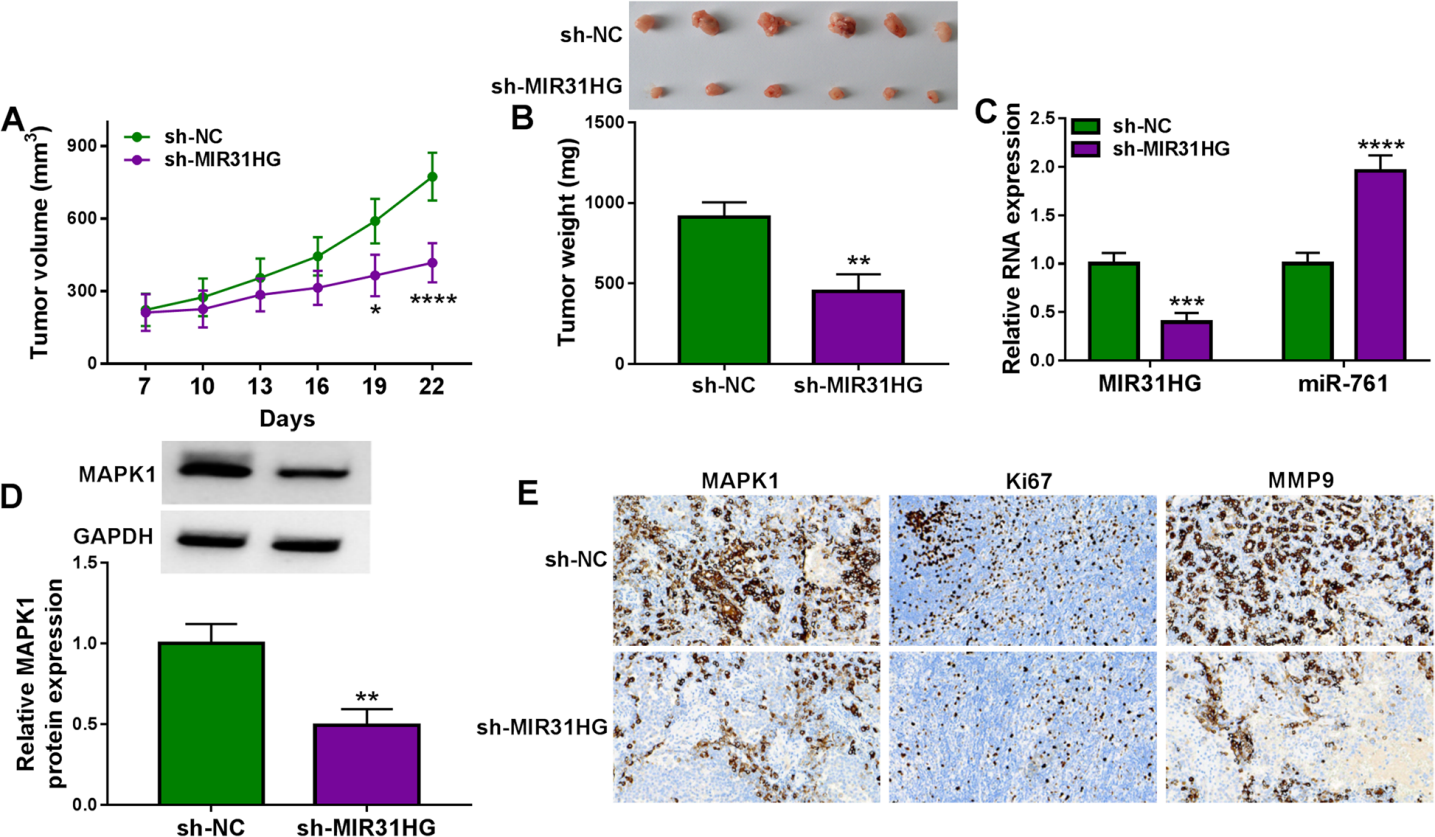
MIR31HG suppression hinders the growth of xenograft tumors. **A** Growth curves of the subcutaneous xenografts produced by sh-MIR31HG-infected or sh-NC-transduced TPC-1 cells (*n* = 6 per group). **B** Representative images and average weight of the subcutaneous xenografts formed by TPC-1 cells transduced with sh-MIR31HG or sh-NC (day 22, *n* = 6 per group). **C** Effects of the transduction of sh-MIR31HG and sh-NC on MIR31HG and miR-761 expression in the subcutaneous xenografts (day 22, *n* = 6 per group) by qRT-PCR, normalized to β-actin or U6 expression. **D** Effects of the transduction of sh-MIR31HG and sh-NC on MAPK1 protein expression in the subcutaneous xenografts (day 22, *n* = 6 per group) by immunoblotting, with GAPDH as a loading control for relative protein quantification. **E** Representative immunohistochemical analysis of tumor sections from subcutaneous xenografts (day 22, *n* = 6 per group) in stained for Ki67, MAPK1, and MMP9. *P < 0.05, **P < 0.01, ***P < 0.001, ****P < 0.0001

Our in vitro findings showed that MIR31HG inhibition causes a growth disadvantage. To further elucidate this observation, we next generated stable depletion of MIR31HG using lentiviral shRNA-MIR31HG (sh-MIR31HG) in TPC-1 cells and injected the cells into the flanks of BALB/c nude mice by subcutaneous injection. Transduction of sh-MIR31HG highly hindered the growth of tumors from TPC-1 cells in subcutaneous xenografts compared with the sh-NC control (Fig. 8A and B). Moreover, sh-MIR31HG-transduced TPC-1 xenografts exhibited reduced expression of MIR31HG and MAPK1 and elevated level of miR-761 (Fig. 8C-E). Growth suppression by MIR31HG depletion was also reflected by a reduction in staining for the proliferation marker Ki67 (Fig. 8E). Our immunohistochemical analysis of tumor sections also showed a decrease in MMP9 staining in MIR31HG-silenced subcutaneous xenografts (Fig. 8E).

## Discussion

It is becoming apparent that deregulation of lncRNAs occurs in thyroid tumorigenesis and tumor progression and such deregulation is crucial for thyroid cancer [10, 17]. Here, we identified MIR31HG as a crucial regulator of thyroid cancer development in vitro and in vivo. Importantly, we demonstrated a novel regulatory mechanism, the miR-761/MAPK1 axis, in the regulation of MIR31HG in thyroid cancer.

A recent report [21] and our data unraveled that MIR31HG expression is at high levels in thyroid cancer. Our findings also suggested the potential of MIR31HG as a biomarker for thyroid cancer prognosis. However, the activity of MIR31HG in thyroid carcinogenesis has not yet been defined. Using loss-of-function phenotype in SW579 and TPC-1 thyroid cancer cells, we first showed that suppression of MIR31HG affects cell growth, motility and apoptosis, indicating the implication of MIR31HG in thyroid tumorigenesis. Furthermore, the main cytoplasmic localization of MIR31HG indicated by our data and the knowledge that lncRNAs participate in gene expression modulation partly through the post-transcriptional mechanism by miRNA competition [23, 25] led us to study the ceRNA crosstalk mediated by MIR31HG.

Previous studies have documented the tumor inhibitory activity of miR-761 in various cancers, such as glioma, osteosarcoma and ovarian carcinoma [19, 28, 35]. Conversely, miR-761 is able to drive the development of gastric cancer, triple-negative breast cancer and NSCLC [8, 22, 30]. The conflicting roles of miR-761 may be at least partly attributed to the different types of tumors and diverse tumor microenvironment. Here, we first uncovered that MIR31HG regulates miR-761 through pairing to miR-761, a low-expressed miRNA in thyroid cancer which has been identified as a strong suppressor in this disease [9, 33]. Importantly, we proved that the effects of MIR31HG suppression on thyroid cancer cells is dependent on increased abundance of miR-761. Similarly, lncRNA HOTAIR upregulates PPME1 to impact thyroid cancer cell behaviors through miR-761 competition [9]. Moreover, lncRNA HOXA11-AS involves the tumorigenesis of thyroid cancer by protecting TRIM29 by inhibiting miR-761 activity [33].

MAPK1 is frequently overexpressed in human tumors and its high levels exert tumorigenic function in numerous cancers, including thyroid cancer [16, 20, 27]. There is a strong suppression of thyroid tumorigenesis upon depletion of MAPK1 in cancer cells [11, 16]. Here, we ascertained MAPK1 as a direct and functionally downstream effector of miR-761. Similarly, several other miRNAs, such as miR-326 and miR-675, target MAPK1 to perform an anti-tumor effect on thyroid cancer [16, 26]. Our findings also established the role of MIR31HG as a post-transcriptional regulator of MAPK1 through the shared binding sequence in miR-761, highlighting the MIR31HG/miR-761/MAPK1 axis in thyroid cancer. The study reported by Wang et al. demonstrated that lncRNA RMRP targeted miR-765 to drive thyroid tumorigenesis through inducing MAPK1 [26].

With our findings, we envision that targeting MIR31HG may have the potential to prevent and inhibit thyroid tumorigenesis. The randomized double-blind placebo-controlled (RDBPC) large-series studies [4] are important for the evaluation of the potential of emerging agents targeting MIR31HG to prevent or inhibit thyroid tumorigenesis in the clinical trial, which will be performed in further work.

## Conclusions

Taken together, these findings have identified the workings of an undescribed regulatory network, in which MIR31HG targets miR-761 to regulate the expression of MAPK1, leading to the alteration of the functional behaviors of thyroid cancer cells. Targeting MIR31HG might be a potential approach for thyroid cancer prevention and treatment.

## Supplementary Information

**Additional file 1: Table S1.** Sequences of qRT-PCR primers.

**Additional file 2.**

## Data Availability

The datasets used or analyzed during the current study are available from the corresponding author on reasonable request.

## Abbreviations

qRT-PCR: Quantitative real-time PCR
lncRNA: Long non-coding RNA
MAPK1: Mitogen-activated protein kinase 1
EdU: 5-Ethynyl-2′-Deoxyuridine
miRNAs: MicroRNAs
ceRNA: Competing endogenous RNA
MTS: 3-(4,5-dimethylthiazol-2-yl)-5-(3-carboxymethoxyphenyl)-2-(4-sulfophenyl)-2H-tetrazolium
MMP9: Matrix metalloproteinase 9
si-MIR31HG: SiRNA-MIR31HG
RDBPC: Randomized double-blind placebo-controlled
