# Supplementary material for: Suppression of MIR31HG affects the functional properties of thyroid cancer cells depending on the miR-761/MAPK1 axis
Source: BMC Endocr Disord. 2022 Apr 20;22:107. doi: 10.1186/s12902-022-00962-3 (PMC9022350; doi:10.1186/s12902-022-00962-3)
Supplement: Supplementary file 1 — Additional file 1: Table S1. Sequences of qRT-PCR primers. [file 12902_2022_962_MOESM1_ESM.docx]

**Supplement Table 1. Sequences of qRT-PCR primers**

| Sequence (5’-3’) | | |
| --- | --- | --- |
| MIR31HG | Forward | AGAGCCCTCAATCACCCACT |
|  | Reverse | ACCAACTCCACACTTTACGTCAT |
| MAPK1 | Forward  Reverse | CCCACCCATATCTGGAGCAG  CCTTCCAATAAGGAGCTTGGA |
| GAPDH | Forward  Reverse | GACAGTCAGCCGCATCTTCT  GCGCCCAATACGACCAAATC |
| miR-761 | Forward | GAGGCAGCAGGGTGAAAC |
|  | Reverse | CTCAACTGGTGTCGTGGA |
| β-actin | Forward | CTCGCCTTTGCCGATCC |
|  | Reverse | GGGGTACTTCAGGGTGAGGA |
| U6 | Forward | CTCGCTTCGGCAGCACA |
|  | Reverse | AACGCTTCACGAATTTGCGT |
