# Supplementary material for: Suppression of MIR31HG affects the functional properties of thyroid cancer cells depending on the miR-761/MAPK1 axis
Source: BMC Endocr Disord. 2022 Apr 20;22:107. doi: 10.1186/s12902-022-00962-3 (PMC9022350; doi:10.1186/s12902-022-00962-3)
Supplement: Supplementary file 2 — Additional file 2. [file 12902_2022_962_MOESM2_ESM.pdf]

The original western blots of Fig2

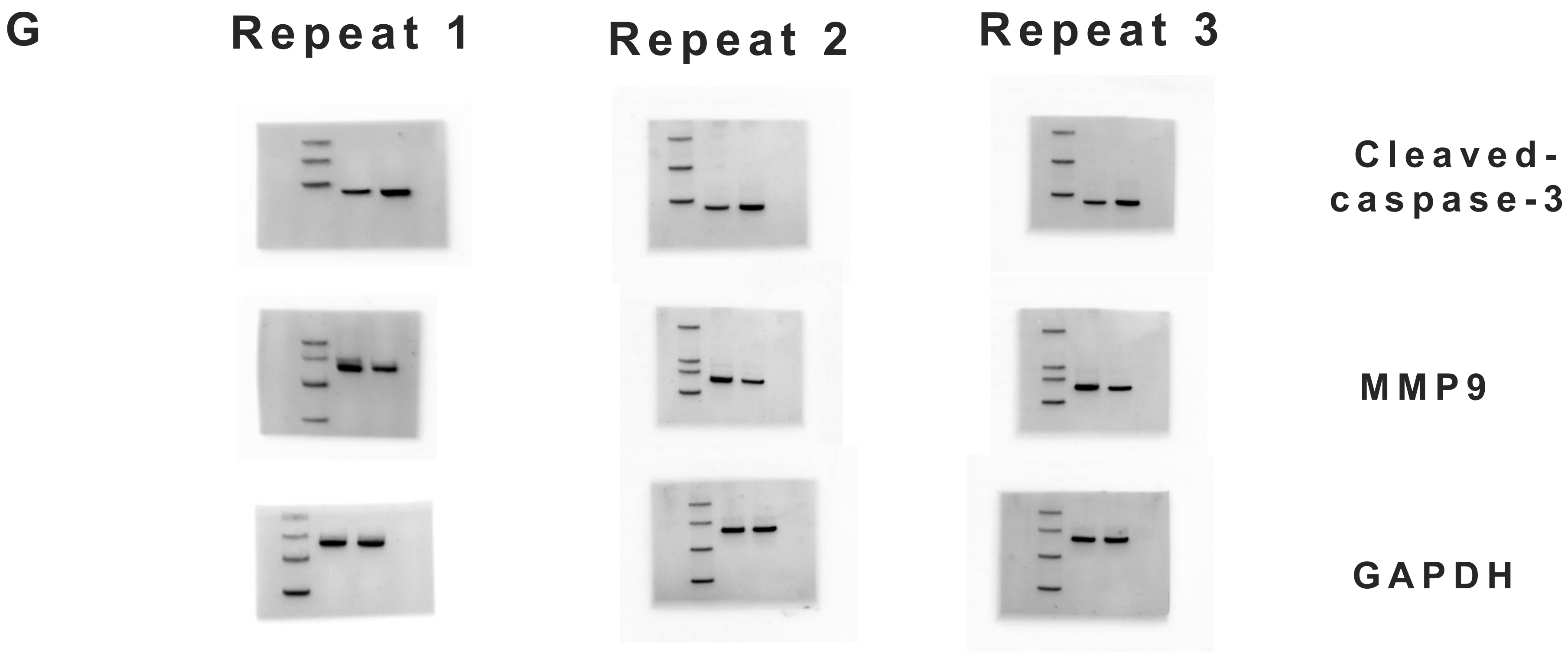

The original western blots of Fig2

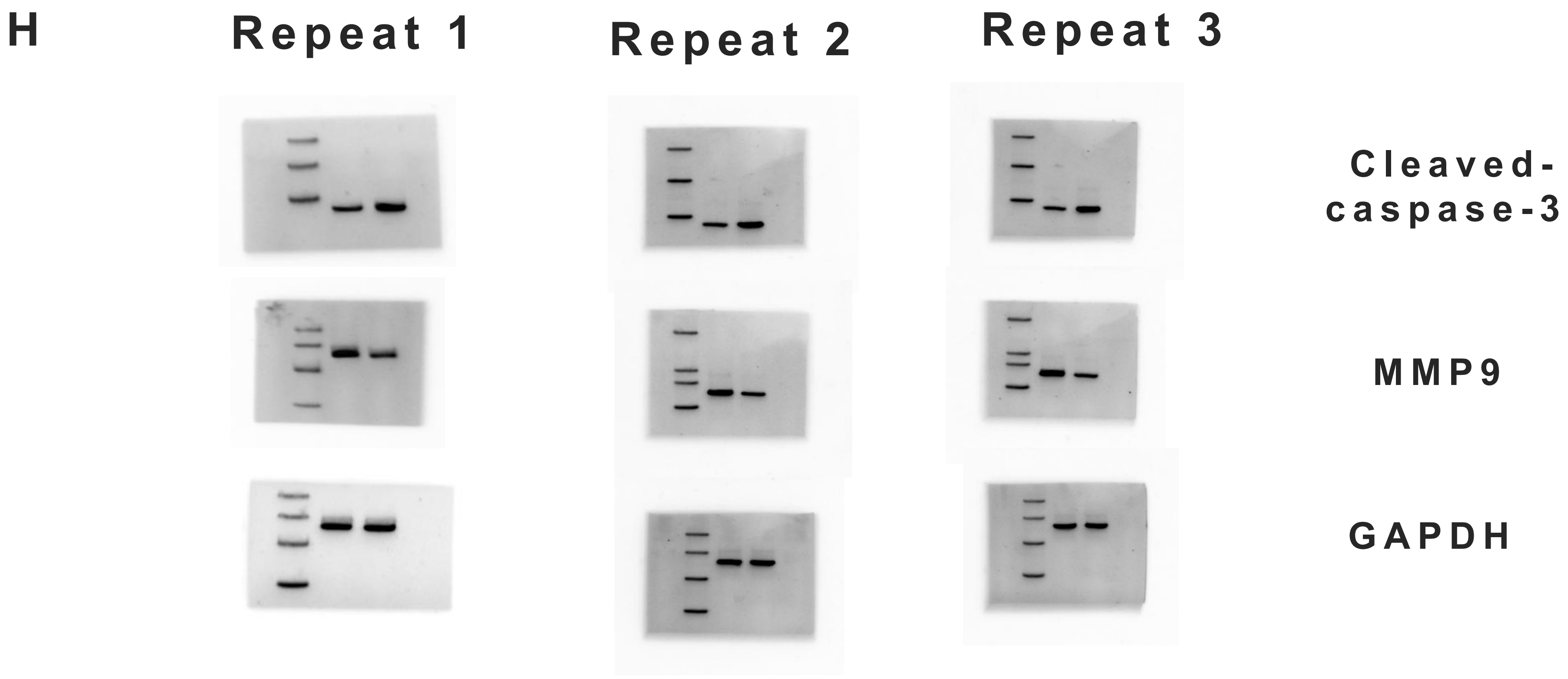

The original western blots of Fig4

G

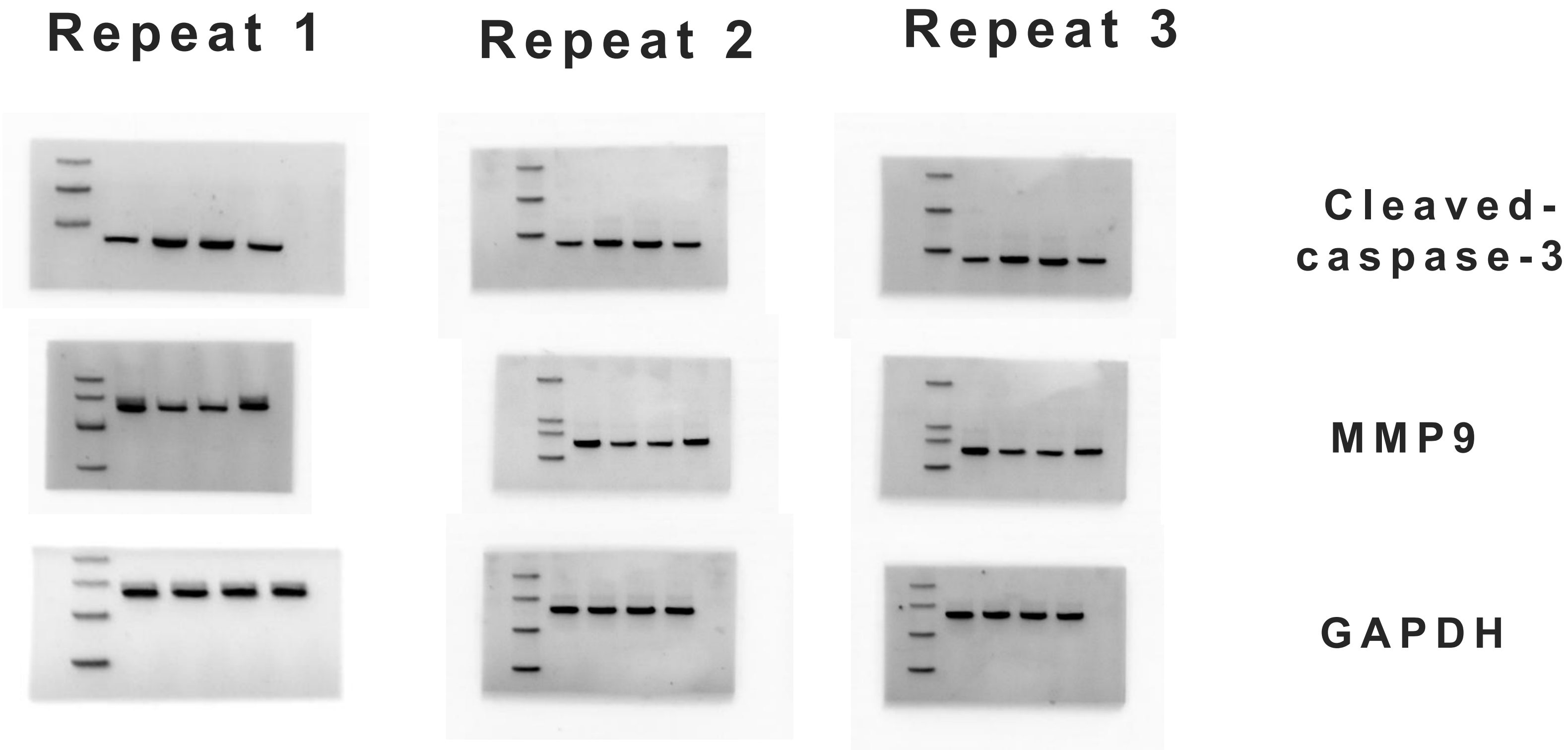

The original western blots of Fig4

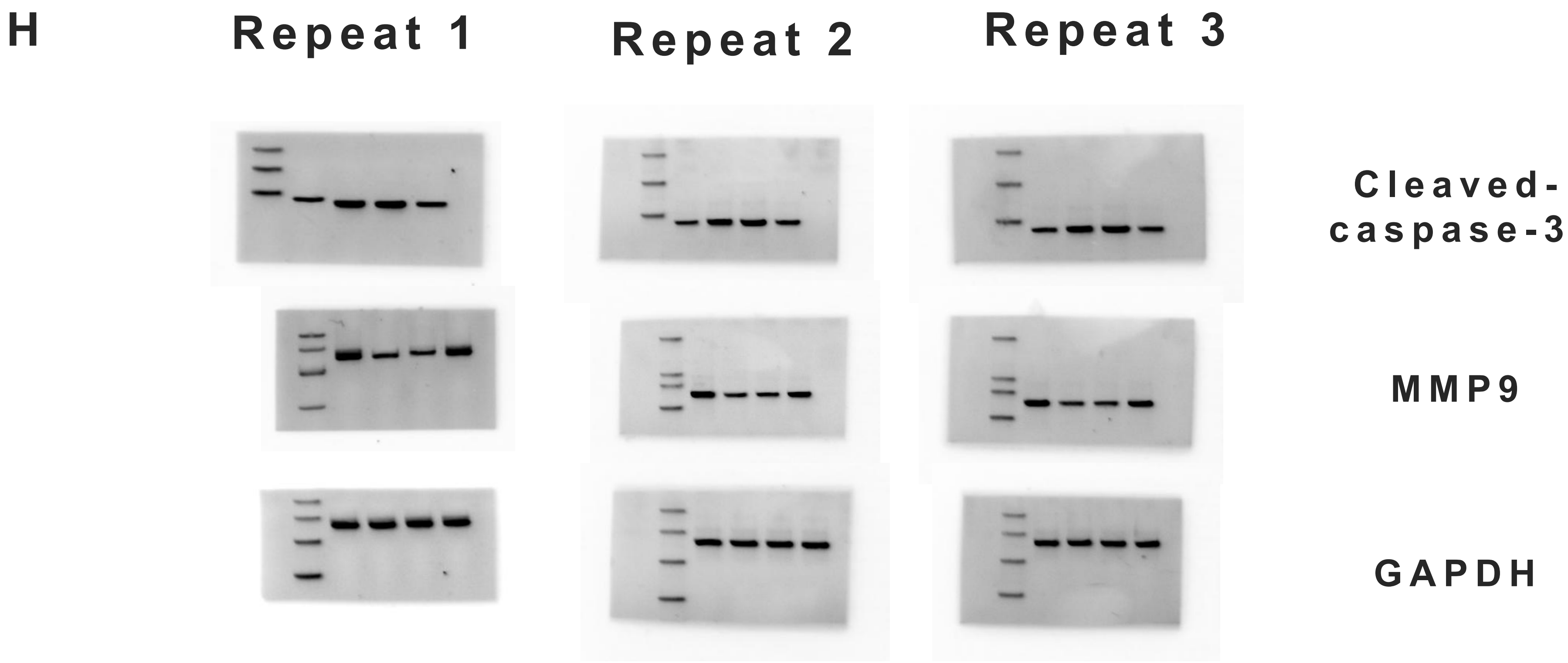

The original western blots of Fig5

F

Repeat 1 Repeat 2 Repeat 3

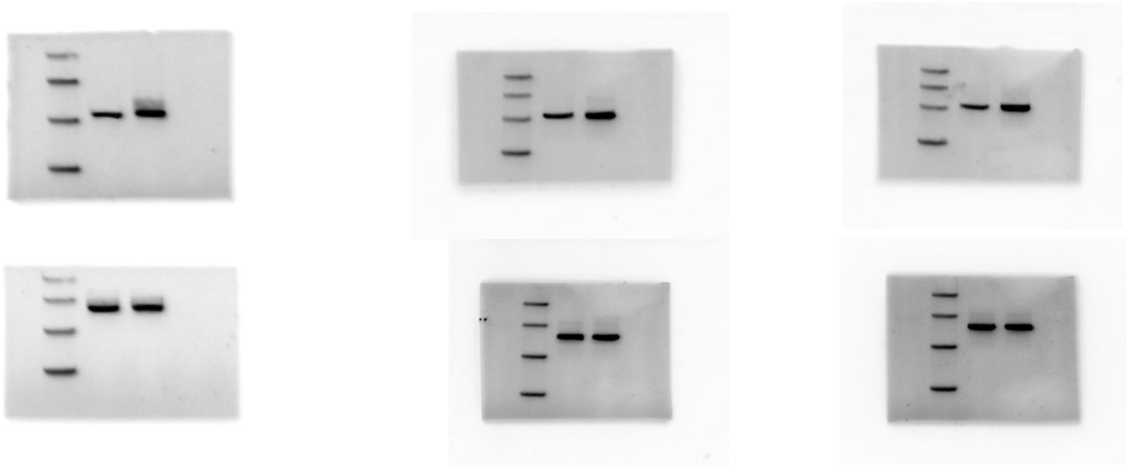

MAPK1

GAPDH

The original western blots of Fig5

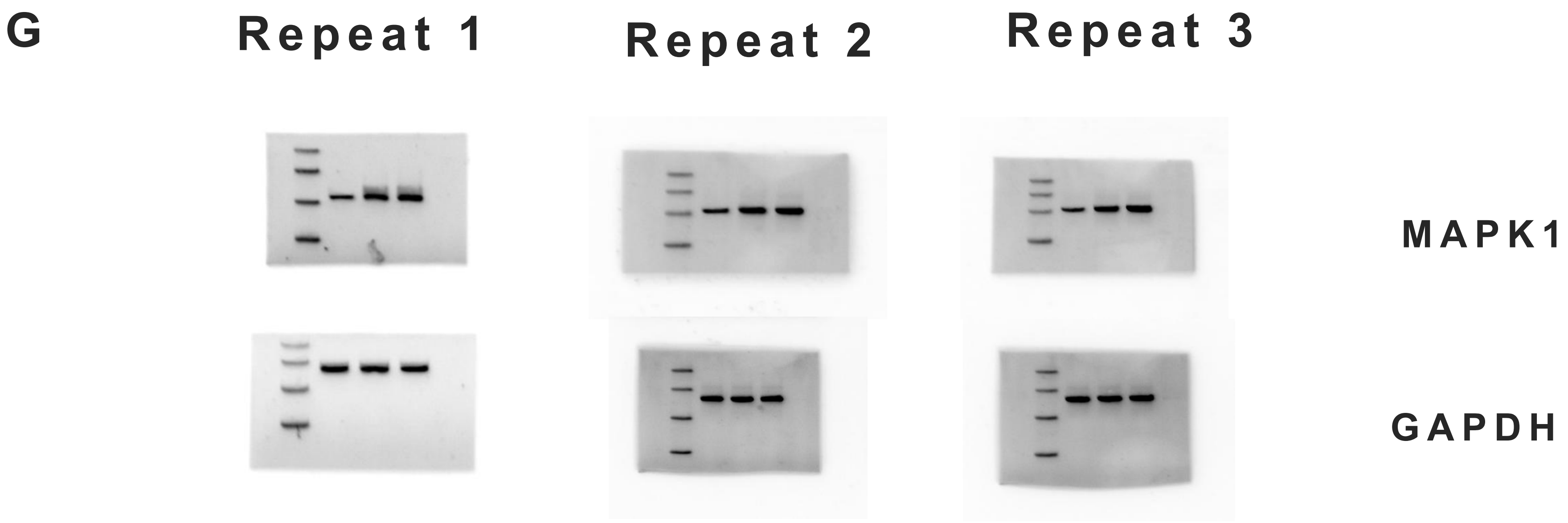

The original western blots of Fig5

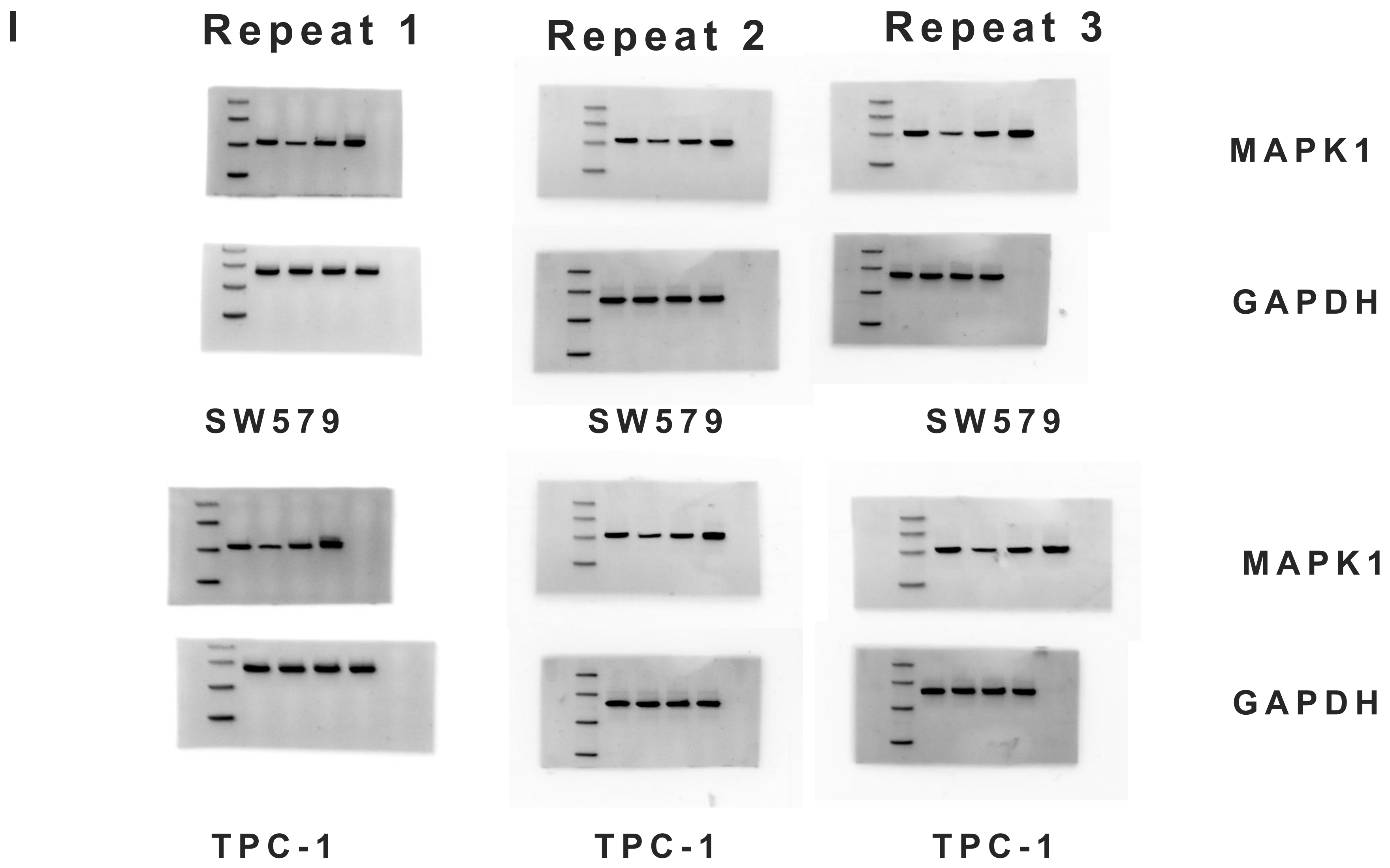

The original western blots of Fig6

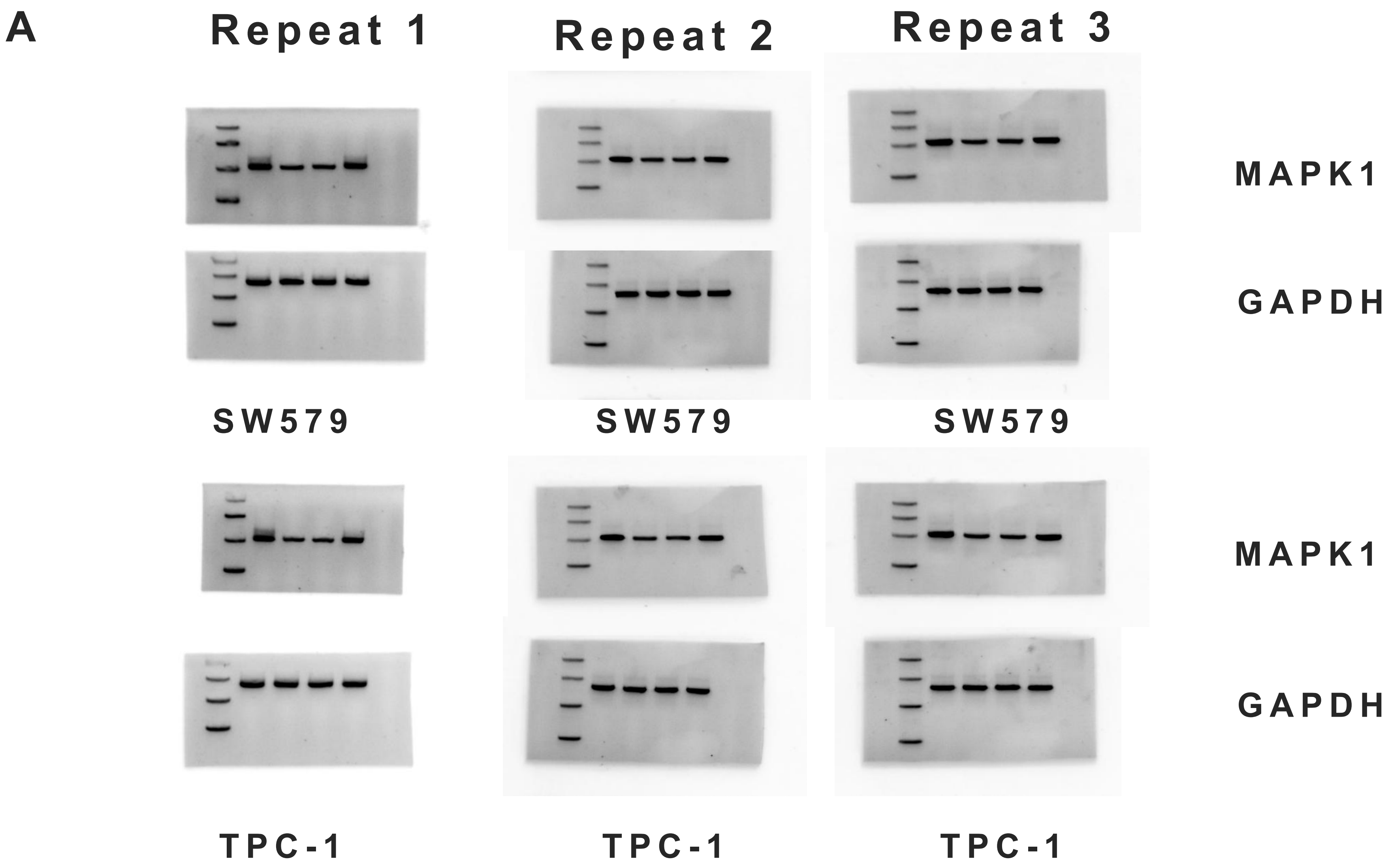

The original western blots of Fig6

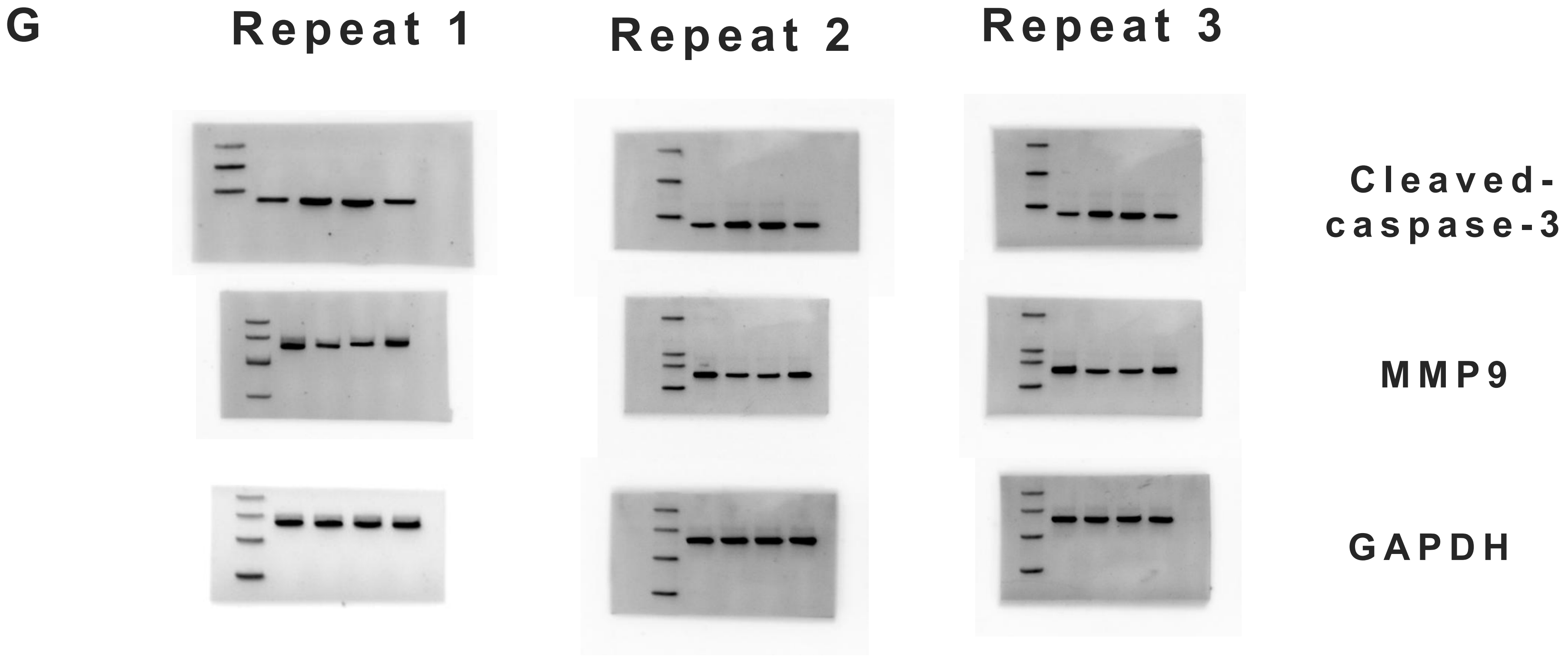

The original western blots of Fig6

H

Repeat 1 Repeat 2 Repeat 3

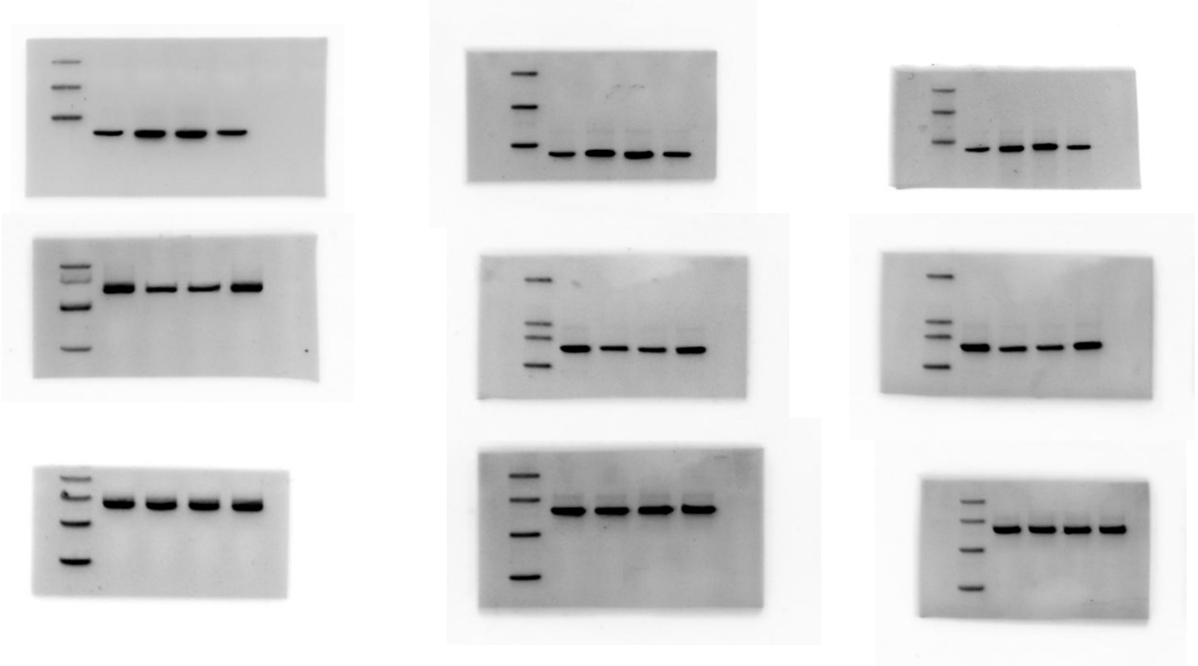

Cleaved-caspase-3

MMP9

GAPDH

The original western blots of Fig7

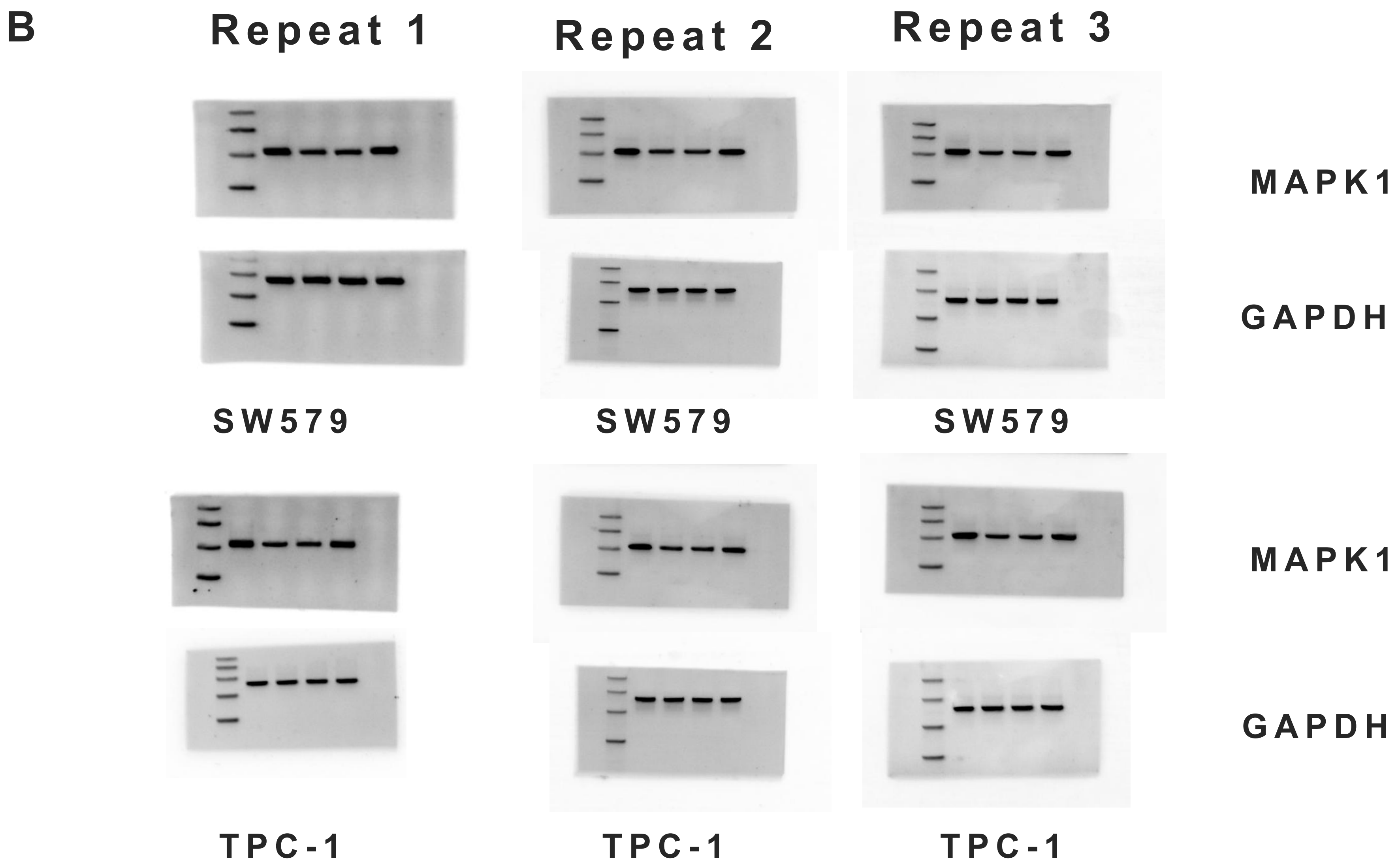

The original western blots of Fig8

D

Repeat 1 Repeat 2 Repeat 3

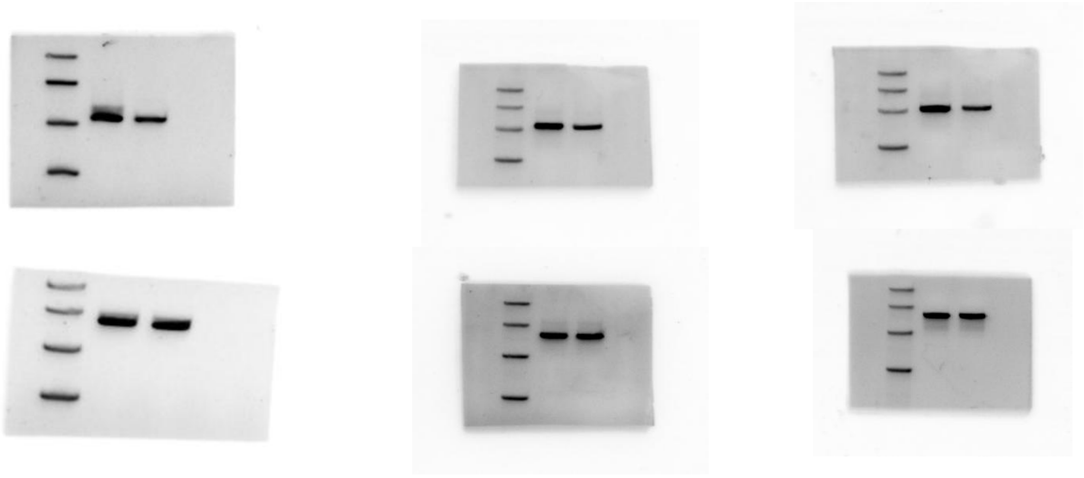

MAPK1

GAPDH
